# Supplementary material for: Comparative analysis of adhesin-like proteins from Methanobacteriales species
Source: Front Microbiol. 2026 Sep 29;17:1897686. doi: 10.3389/fmicb.2026.1897686 (PMC13658579; doi:10.3389/fmicb.2026.1897686)
Supplement: Supplementary file 2 [file Data_Sheet_2.PDF]

Table S1. Metadata on strains and ALPs

Genus	Species	Strain	Source/ accession number
Methanothermobacter	*marburgensis*	Marburg	CP001710.1
Methanosphaera	*stadtmanae*	DSM3091	CP000102.1
Methanobrevibacter	*sp.* AbM4		CP004050.1
Methanosphaera	*sp.* BMS		CP014213.1
Methanothermus	*fervidus*	DSM2088	CP002278.1
Methanobacterium	*formicicum*	BRM9	CP006933.1
Methanosphaera	*sp.* ISO3-F5		CP118753.2
Methanobacterium	*lacus*	AL-21	CP002551.1
Candidatus Methanobrevibacter	*massiliense*	Vir-13MRS	JARBXM010000000
Methanobacterium	*sp.* MB1		HG425166
Methanobrevibacter	*millerae*	SM9	CP011266.1
Methanobrevibacter	*olleyae*	YLM1	CP014265.1
Methanobacterium	*paludis*	SWAN1	CP002772.1
Methanobrevibacter	*ruminantium*	M1	CP001719.1
Methanothermobacter	*thermautotrophicus*	Delta H	AE000666.1
Methanobrevibacter	*smithii*	P15MS	https://github.com/SDMUG/Methanobrevibacter_Enrichment/blob/main/Nanopore%20Sequencing/contigs/P15MS_consensus.fasta
Methanobrevibacter	*smithii*	P31	https://github.com/SDMUG/Methanobrevibacter_Enrichment/blob/main/Nanopore%20Sequencing/contigs/P31_consensus.fasta
Methanobrevibacter	*smithii*	P48	https://github.com/SDMUG/Methanobrevibacter_Enrichment/blob/main/Nanopore%20Sequencing/contigs/P48_consensus.fasta
Methanobrevibacter	*smithii*	P86 BT	https://github.com/SDMUG/Methanobrevibacter_Enrichment/blob/main/Nanopore%20Sequencing/contigs/P86_BT_consensus.fasta
Methanobrevibacter	*smithii*	P88	https://github.com/SDMUG/Methanobrevibacter_Enrichment/blob/main/Nanopore%20Sequencing/contigs/P88_consensus.fasta
Methanobrevibacter	*smithii*	KB11	CP017803.
Methanobrevibacter	*smithii*	CE91-St68	AP025587.1
Methanobrevibacter	*smithii*	ATCC 35061	CP000678
Methanobrevibacter	*intestini*	WWM1085	NQLD00000000
Methanobrevibacter	*intestini*	TLL-48-HuF1	CP081485.
Methanobrevibacter	*intestini*	P89	https://github.com/SDMUG/Methanobrevibacter_Enrichment/blob/main/Nanopore%20Sequencing/contigs/P89_consensus.fasta
Methanobrevibacter	*intestini*	P82MpT1	https://github.com/SDMUG/Methanobrevibacter_Enrichment/blob/main/Nanopore%20Sequencing/contigs/P82MpT1_sans_C_consensus.fasta
Methanobrevibacter	*intestini*	P45	https://github.com/SDMUG/Methanobrevibacter_Enrichment/blob/main/Nanopore%20Sequencing/contigs/P45_consensus.fasta
Methanobrevibacter	*intestini*	G0370 i3	CP187956.1
