# Supplementary material for: Comparative analysis of adhesin-like proteins from Methanobacteriales species
Source: Front Microbiol. 2026 Sep 29;17:1897686. doi: 10.3389/fmicb.2026.1897686 (PMC13658579; doi:10.3389/fmicb.2026.1897686)
Supplement: Supplementary file 3 [file Data_Sheet_3.PDF]

Table S2. ALP and ALP class distribution in genomes

Species	genome size	total protein-coding genes	percentage of genes	percentage of genome	ALP class IA	ALP class IB	ALP class IIA	ALP class IIB	ALP class Other
Methanobacterium lacus AL-21	2583753	2512	2.0	4.5	46.9	0.0	8.2	8.2	18
Methanobacterium formicicum BRM9	2449988	2352	0.7	1.5	75.0	0.0	0.0	6.3	3
Methanobacterium paludis SWAN-1	2546541	2339	1.0	2.8	29.2	8.3	0.0	12.5	12
Methanobacterium sp . Mb1	2029766	2021	0.3	0.6	28.6	0.0	71.4	0.0	0
Methanobrevibacter intestini TLL-48-HuF1	1951395	1777	3.8	13.7	25.4	20.9	9.0	14.9	20
Methanobrevibacter massiliense Vir-13MRS	1834388	1870	2.1	6.7	17.9	20.5	7.7	20.5	13
Methanobrevibacter millerae SM9	2543538	2269	3.6	14.6	19.8	9.9	25.9	4.9	32
Methanobrevibacter olleyae YLM1	2201192	1834	3.3	7.0	38.3	6.7	13.3	10.0	19
Methanobrevibacter ruminantium M1	2937203	2154	4.8	15.1	20.4	11.7	14.6	16.5	38
Methanobrevibacter smithii PS	1853160	1795	2.7	10.5	22.9	25.0	14.6	8.3	14
Methanobrevibacter sp . AbM4	1998189	1671	1.9	4.7	19.4	16.1	12.9	6.5	14
Methanosphaera sp . BMS	2868093	2108	6.3	19.6	40.9	12.1	6.8	10.6	39
Methanosphaera sp . ISO3-F5	2506044	2281	1.8	11.2	45.2	2.4	16.7	0.0	15
Methanosphaera stadtmanae DSM3091	1767403	1534	2.7	10.4	57.1	11.9	4.8	0.0	11
Methanothermobacter marburgensis Marburg	1639135	1752	0.6	1.4	18.2	0.0	18.2	9.1	6
Methanothermobacter thermautotrophicus DeltaH	1751377	1802	0.5	1.3	11.1	0.0	11.1	0.0	7
Methanothermus fervidus V24S	1243342	1311	0.8	1.6	45.5	0.0	9.1	0.0	5
